# Corn Kernel Segmentation and Damage Detection Using a Hybrid Watershed–Convex Hull Approach

**DOI:** 10.3390/foods15020404

**Published:** 2026-01-22

**Authors:** Yi Shen, Wensheng Wang, Xuanyu Luo, Feiyu Zou, Zhen Yin

**Affiliations:** 1 College of Mechanical and Electrical Engineering, Beijing Information Science and Technology University, Beijing 102206, China; yishen@bistu.edu.cn (Y.S.); xy_luo@bistu.edu.cn (X.L.); 2023010006@bistu.edu.cn (F.Z.); 2023010244@bistu.edu.cn (Z.Y.); 2 Key Laboratory of Modern Measurement & Control Technology, Ministry of Education, Beijing Information Science and Technology University, Beijing 100192, China

**Keywords:** sticky corn kernel, image segmentation, damage detection, watershed and convex hull-SVM classification, food quality control

## Abstract

Accurate segmentation of adhered (sticky) corn kernels and reliable damage detection are critical for quality control in corn processing and kernel selection. Traditional watershed algorithms suffer from over-segmentation, whereas deep learning methods require large annotated datasets that are impractical in most industrial settings. This study proposes W&C-SVM, a hybrid computer vision method that integrates an improved watershed algorithm (Sobel gradient and Euclidean distance transform), convex hull defect detection and an SVM classifier trained on only 50 images. On an independent test set, W&C-SVM achieved the highest damage detection accuracy of 94.3%, significantly outperforming traditional watershed SVM (TW + SVM) (74.6%), GrabCut (84.5%) and U-Net trained on the same 50 images (85.7%). The method effectively separates severely adhered kernels and identifies mechanical damage, supporting the selection of intact kernels for quality control. W&C-SVM offers a low-cost, small-sample solution ideally suited for small-to-medium food enterprises and breeding laboratories.

## 1. Introduction

Image processing has revolutionized agricultural quality assessment, particularly for maize, a vital staple crop. Precise evaluation of maize kernel morphology is essential for ensuring kernel quality, which directly impacts yield and nutritional value. The morphological characteristics of maize kernel two-dimensional images reflect their quality attributes, serving as vital assessment tools for producers and markets. However, adhered kernels pose significant challenges during image processing. In recent years, computer vision techniques have been progressively applied to the automated measurement of maize ear traits. Yet, certain measurement methods require meticulously arranged ears, thereby reducing efficiency [1,2]. Failure to accurately segment adhered kernels may lead to erroneous detection results, compromising quality assessments, posing health risks and causing economic losses [3,4]. Consequently, developing and implementing precise image segmentation algorithms is essential to provide accurate data for damage detection and enhance market corn quality.

Based on the current state of visual inspection research in maize breeding, widely applied methods for separating adhered kernels include image processing-based morphological operations, watershed algorithms and convex hull defect algorithms [5]. Image segmentation—the technique and process of extracting targets of interest based on features such as greyscale, color, texture and geometry—constitutes a critical step in transitioning from image processing to image analysis. It remains both a technical challenge and a research hotspot [6,7]. Watersheding represents a classical image segmentation approach, wherein the image is conceptualized as a topological terrain. This topography is partitioned into drainage basins associated with local minimum values [8,9]. Convex Hull Defects Analysis involves identifying defective regions within a graphic by calculating the difference between its convex hull (minimum convex polygon) and the actual contour. RA Ketcham [10] introduced a novel watershed method for separating and characterizing discrete objects within 3D datasets. Deep learning-based algorithms have also gained prominence in maize kernel image analysis, with their robust feature learning capabilities driving new breakthroughs in image analysis [11,12]. Qing Geng et al. [13] proposed an image segmentation model termed TransUNet to achieve kernel segmentation in wheat ears. However, these approaches still exhibit issues such as insufficient segmentation accuracy and poor adaptability to complex backgrounds when processing intricate adhered maize kernel images. For sticky corn kernels, the irregular adhesion (e.g., partial overlap) and similar grayscale to background (e.g., light reflection on kernel surfaces) make traditional methods (e.g., U-Net) require large annotated datasets (≥1000 images), which is impractical for maize breeding labs with limited sampling resources.

Given this research landscape, this paper focuses on the segmentation of adhered maize kernel images and the analysis of damaged patterns. On one hand, it comprehensively employs morphological processing, watershed algorithms and convex hull defect segmentation. Morphological operations, including opening and closing, are applied to reduce noise and fill small holes in binary kernel images. The watershed algorithm excels at preliminary separation of adhered regions, while convex hull defects precisely locate minute adhesion points. Together, they form a complementary ‘coarse segmentation fine optimization’ logic. This approach requires minimal annotated data, making it suitable for agricultural scenarios with small sample sizes to achieve accurate segmentation of adhered corn kernels. Concurrently, by extracting shape parameters such as area, perimeter, circularity and rectangularity from corn kernels, and integrating these with SVM model analysis, a comprehensive assessment of damage severity is achieved [14,15,16,17].

The innovations of this study are primarily reflected in three aspects: Firstly, an improved watershed algorithm with Sobel gradient optimization (reducing over-segmentation) and Euclidean distance transformation (enhancing cohesive localization), this reduces over-segmentation errors that previously led to miss election of high-viability kernels, directly supporting high-yield breeding; Secondly, a “coarse segmentation (watershed)—fine optimization (convex hull defects)” framework specifically designed for sticky corn kernels. tailored to sticky corn (valued for its high starch content, a key nutritional trait), ensuring intact, nutritionally rich kernels are accurately identified; Thirdly, a low-sample support vector machine damage detection model suitable for maize breeding. These innovations aim to provide more precise and reliable technical support for maize kernel quality assessment.

Accurate segmentation and damage assessment of sticky corn kernels can help remove defective grains and support the selection of intact corn kernels. This is an important indicator of high-quality food ingredients.

## 2. Materials and Methods

The experimental approach integrates advanced image processing techniques to address the challenges of segmenting adhered maize kernels and assessing damage. Accurate segmentation is essential for ensuring the quality of maize kernels, which directly impacts kernel viability and nutritional quality. The approach combines the watershed algorithm for preliminary segmentation, convex hull defect detection for precise boundary correction and morphological operations for noise reduction. These methods are complemented by feature extraction techniques that capture essential kernel characteristics, such as area, perimeter and elongation, which are crucial for assessing kernel damage [18,19,20,21]. A support vector machine (SVM) classifier then analyzes these features to determine the extent of damage. This multi-step process ensures high-precision segmentation and classification, providing reliable results even with limited sample sizes, thus supporting efficient maize breeding and quality control for high-yield and nutritionally superior crops.

### 2.1. Sticky Corn Kernel Segmentation System Framework

**Figure 1:**
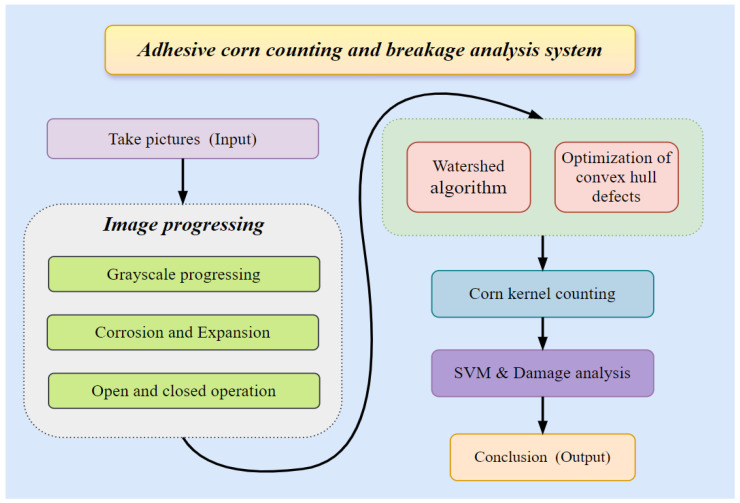
Adhesive corn counting and breakage analysis system.

The system framework for dense adhesion corn kernel segmentation and damage rate detection designed in this study is shown in Figure 1, comprising six modules: image acquisition, preprocessing, adhesion segmentation, feature extraction, damage analysis and model training. The system captures corn kernels using a high-resolution camera, processes them through preprocessing and then employs an improved watershed algorithm combined with convex hull defect detection to segment adhered kernels. Morphological features of the kernels are extracted, and damage rate detection is ultimately achieved through feature analysis. Each module is designed to link kernel segmentation results to kernel quality for high-yield, high-nutrition breeding: the damage analysis module focuses on kernel intactness as a key quality indicator.

### 2.2. Corn Kernel Samples and Image Acquisition

Sticky corn kernels were dried to 13.5 ± 0.5% moisture content (wet basis) and stored at 4 °C until imaging. Images were acquired using a dedicated vision system designed for repeatable food raw material inspection. The setup consisted of a Basler acA2500-14uc color camera (2592 × 1944 pixels, 1/1.2″ sensor, 15 fps) equipped with a Computar M1214-MP2 12 mm F1.4 lens (field of view ≈ 30°). Uniform illumination was provided by an OPT-RL100 ring light (12 W, 5000 K, 800 ± 50 lux, uniformity ≥90%) mounted 30 cm above a 3 mm-thick black matte PVC background plate to minimize specular reflections on kernel surfaces. All experimental equipment is sourced from Beijing, China. For each acquisition, 80–150 kernels were randomly scattered on the background to induce natural adhesion without manual arrangement.

### 2.3. Image Preprocessing

To reduce environmental noise and improve the accuracy of subsequent research and data analysis by mitigating factors such as uneven light sources and non-smooth backgrounds, preprocessing the original image yields grayscale and binary images. The image preprocessing process primarily includes three key steps: grayscale conversion, binarization and morphological processing. The dataset consisted of 50 images of sticky corn kernels acquired from a single physical batch (harvested on the same date, with natural variability in adhesion and damage arising from random scattering and imaging conditions). Each image contained 80–150 randomly scattered kernels, inducing natural adhesion under controlled laboratory conditions. Manual annotation was performed by two research team members to delineate individual kernel boundaries and classify each as intact or damaged based on visible mechanical defects. For SVM training and evaluation, the 50 annotated images were split at the image level into a training set of 40 images (80%) and an independent test set of 10 images (20%). To prevent data leakage, images were randomly assigned to sets with no overlap between training and testing.

#### 2.3.1. Grayscale Conversion

**Figure 2:**
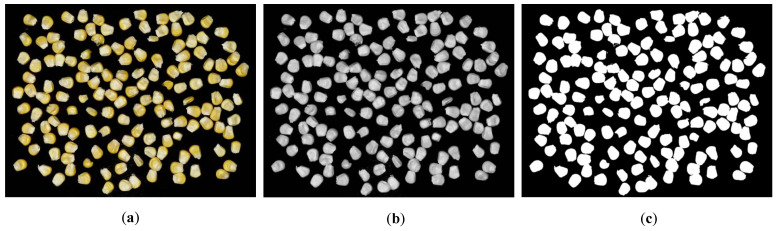
Preprocessed image: (**a**) Original-image; (**b**) Grayscale-image; (**c**) Binarized-image.

The original image captured through imaging is an RGB format Figure 2a, which follows the additive color model. For corn kernel adhesion images, color analysis proves unnecessary. By reducing the raw data volume, we can lower the complexity of subsequent research. Therefore, the image is converted to grayscale Figure 2b. The grayscale calculation employs floating-point algorithms as follows:

(1) Gray=0.299×R+0.587×G+0.114×B

*R*—red channel pixel value; *G*—green channel pixel value; *B*—blue channel; pixel value.

#### 2.3.2. Binary Processing

In image segmentation of adhered corn kernels, binary processing primarily converts images to black and white for better differentiation between kernels and background. The results of this process are shown in Figure 2c. By setting a threshold, the processed grayscale image’s pixels are divided into two categories: one representing corn kernels and the other representing background. This simplifies image information, highlights kernel contours and lays the foundation for subsequent segmentation and feature extraction. Our study employs the Otsu method [22], also known as the maximum inter-class variance (IVC) approach. The algorithm aims to find an optimal threshold that maximizes the difference between foreground and background while minimizing intra-class variations. Inter-class variance measures the disparity between categories, with the highest variance indicating optimal separation. For grayscale values ranging from [0, *L* − 1] (where *L* is the number of gray levels), the probability $p_{i}$ for a pixel with gray value *i* is calculated as: $n_{i}$ where *N* represents the total number of pixels in the image:

(2) $$p_{i}=\frac{n_{i}}{N};\sum_{i=0}^{L-1}p_{i}=1$$

Assume a threshold t divides the image into foreground and background, where the gray value range of the foreground is [0, *t*] and that of the background is [*t* + 1, *L* − 1]. The probability $\omega_{1}(t)$ of foreground and probability $\omega_{2}(t)$ of background:

(3) $$\omega_{1}(t)=\sum_{i=0}^{t}p_{i};\omega_{2}(t)=\sum_{i=t+1}^{L-1}p_{i}=1-\omega_{1}(t)$$

The average gray value $\mu_{1}(t)$ of the foreground and the average gray value $\mu_{2}(t)$ of the background:

(4) $$\mu_{1}(t)=\frac{\sum_{i=0}^{t}i\times p_{i}}{\omega_{1}(t)};\mu_{2}(t)=\frac{\sum_{i=t+1}^{L-1}i\times p_{i}}{\omega_{2}(t)}$$

Global average gray value of the image μ:

(5) $$\mu =\sum_{i=0}^{L-1}i\times p_{i}=\omega_{1}(t)\times \mu_{1}(t)+\omega_{2}(t)\times \mu_{2}(t)$$

Class inter variance $\sigma_{B}^{2}(t)$:

(6) $$\sigma_{B}^{2}(t)=\omega_{1}(t)\times {\left(\mu_{1}(t)-\mu \right)}^{2}+\omega_{2}(t)\times {\left(\mu_{2}(t)-\mu \right)}^{2}$$

The OTSU method is to find a threshold *t* so that the inter-class variance $\sigma_{B}^{2}(t)$ reaches the maximum value:

(7) $$t_{optimal}=\arg\underset{0\leq t\leq L-1}{\max}\sigma_{B}^{2}(t)$$

This value is the optimal threshold. Compared with the fixed threshold method and other binary processing methods, OTSU is simpler and more effective, which can automatically determine the optimal threshold without manual intervention, improving accuracy and efficiency.

#### 2.3.3. Morphological Treatment

The primary purpose is “denoising,” which involves eliminating fine defects in images to achieve cleaner visuals. In the adhesion image analysis under study, morphological processing mainly includes operations such as erosion and dilation, as well as open and closed operations. Erosion and dilation are complementary techniques. Erosion gradually reduces and refines high-contrast areas or white regions in the original image, effectively narrowing foreground areas and eliminating minor noise. This process can be implemented through convolution operations using predefined structural elements with the original image. Dilation, conversely, expands high-contrast or white regions to identify maximum regions in the image, also achieved through convolution operations using predefined structural elements.

• Erosion: The structural element *B* erodes the image *A*, which is written as A⊖B:
  (8) $$A⊖B=\left\{z\left|{\left(B\right)}_{z}\subseteq A\right.\right\}$$
  ${\left(B\right)}_{z}$ represents the set of structural elements *B* after translation by *z*.
• Expanding: The structural element B expands the image A, which is written as A⊕B
  (9) $$A⊕B=\left\{z\left|{\left(\overset{⌢}{B}\right)}_{z}\cap A\neq \emptyset \right.\right\}$$
  $\overset{⌢}{B}$ is a reflection of *B* (symmetrical about the origin).

Widely used in mathematical morphology for image processing, the opening operation and closing operation are mutually complementary techniques that play pivotal roles in refining image quality. The opening process commences by applying erosion, a morphological transformation that utilizes a predefined structuring element to selectively eliminate small, unwanted bright noise pixels—such as salt-and-pepper noise—both within and outside the contours, while naturally reducing the overall image size. Following this noise-removal step, dilation is applied to the eroded image: this second transformation expands the remaining structural elements to restore the image to its original spatial dimensions, ensuring that key features of the target objects are not distorted or diminished.

**Figure 3:**
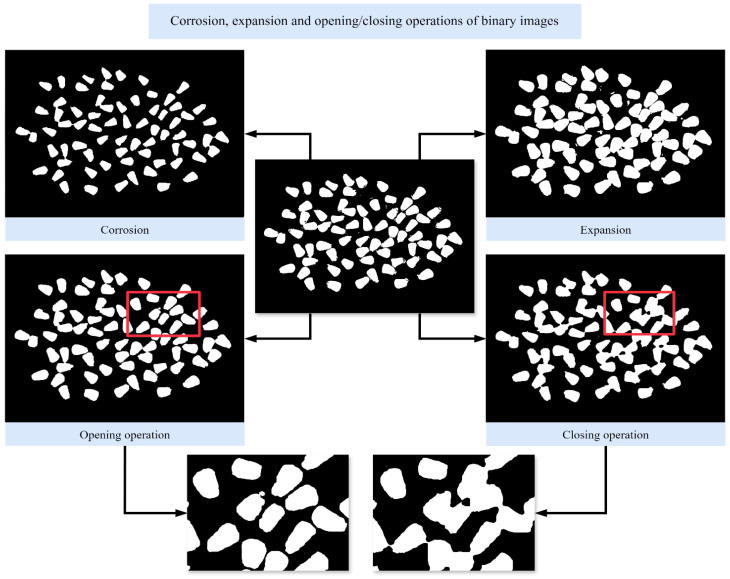
Corrosion, expansion and opening/closing operations of binary images. Perform corrosion, expansion, opening and closing operations on a binary image separately. The two enlarged images below can illustrate the difference in processing results between open and closed operations.

The closing operation, by contrast, operates in the reverse sequence of the opening operation. It first employs dilation to fill in tiny dark cavities (small black holes or pinholes) within the foreground regions and along the contours, which often arise from uneven lighting or minor imaging artifacts. Subsequently, erosion is performed to shrink the slightly expanded image back to its approximate original scale, effectively sealing small defects outside the contours without altering the main object shape. Together, these two complementary operations balance noise reduction and shape preservation, making them indispensable for image preprocessing in computer vision tasks like object detection and segmentation. Refer to Figure 3 for the preprocessing method.

• The opening formula is written as A∘B:
  (10) A∘B=(A⊖B)⊕B
• Closed operations are written as A•B:
  (11) A•B=(A⊕B)⊖B

### 2.4. Image Adhesion Segmentation

This study employs a watershed algorithm and convex hull defect detection for adhesion corn kernel segmentation, with complementary advantages enabling high-precision counting. The watershed algorithm [23], a topological-based morphological segmentation method, separates adhered objects by simulating water diffusion in gradient images. Convex hull, a concept relative to point sets, defines a subset of points that forms a convex polygon completely enclosing all points [24]. By identifying both convex hulls and concave regions in target contours, the convex hull defect detection method effectively detects adhesion boundaries. Their combined application significantly enhances segmentation accuracy, resulting in the Watershed and Convex Hull Defects-SVM (W&C-SVM) approach, where SVM serves as the machine learning algorithm for subsequent model training.

#### 2.4.1. The Watershed Algorithm

Essentially a mathematical morphological method rooted in topological theory, its core principle originates from the watershed concept in geodesy. By treating images as topographic landscapes and grayscale images as terrain surfaces where pixel brightness values correspond to elevation, it simulates water diffusion on gradient images to achieve target segmentation. The algorithm detects local minima as initial markers, then constructs watershed lines based on gradient information to separate contiguous objects. In this study, the grayscale image of corn kernels demonstrates significant contrast between contour targets and background gradient distributions. The Watershed Algorithm effectively identifies and extracts regional boundaries, enabling cohesive segmentation of corn kernels. Within the watershed algorithm, the image gradient is first computed. This study employs the Sobel operator as the gradient operator. For a pixel point (*x*, *y*) in the image, its gradient in the x and y directions is as follows:

(12) $$G_{x}(x,y)=\sum_{i=-1}^{1}\sum_{j=-1}^{1}k_{ij}f(x+i,y+j)$$

(13) $$G_{y}(x,y)=\sum_{i=-1}^{1}\sum_{j=-1}^{1}l_{ij}f(x+i,y+j)$$

$k_{ij}$ is the Sobel template coefficient in the *x* direction; $l_{ij}$ is the Sobel template coefficient in the *y* direction.

(14) $$G(x,y)=\sqrt{G_{x}{\left(x,y\right)}^{2}+G_{y}{\left(x,y\right)}^{2}}$$

This study employs a 3 × 3 Sobel operator to compute gradients in the *x*/*y* directions, normalizes gradient magnitudes (range 0–255) and applies Gaussian filtering (*σ* = 1.5) to smooth the gradient image, thereby reducing misclassification caused by edge noise.

The second step in the watershed algorithm involves distance transformation, which calculates the distance between each image pixel and its nearest background pixel. Using Euclidean transformation, for a binary image *B*(*x*, *y*) (where background pixels have value 0 and foreground pixels 1), the distance transformation value *D*(*x*, *y*) is defined as the minimum distance between the current pixel and all background pixels.

(15) $$D(x,y)=\underset{(x^{\prime },y^{\prime })\in B}{\min}\sqrt{{\left(x-x^{\prime }\right)}^{2}+{\left(y-y^{\prime }\right)}^{2}}$$

Following preprocessing such as gradient computation and distance transformation, a marked image *M*(*x*, *y*) is obtained. A watershed transformation is then applied to identify watershed lines. The watershed algorithm simulates the process of water overflowing terrain and can be expressed as follows:

(16) $$W=\left\{(x,y)\in M\left|h(x,y)=\underset{p\in C(x,y)}{\max}h(p)\right.\right\}$$

*W* is the watershed line, which is the height value of a pixel (e.g., gradient amplitude or distance transformation value) and is the set of all pixels that can be reached from a pixel.

#### 2.4.2. Convex Hull Defect Detection

For a given set of points on a two-dimensional image, the convex hull denotes the smallest convex polygon encompassing all these points. In image processing, for an object with a complex shape, the convex hull represents the smallest convex region that fully encloses the object. Convex hull defect detection calculates defect locations by measuring discrepancies between the object’s contour and its convex hull. Based on the analysis of the contour point set, the positions of contour points and their corresponding points on the convex hull are compared to identify points deviating from the convex hull. The regions formed by these points constitute convex hull defects. Given an edge *AB* of the convex hull and a point *C* on the contour, the distance (i.e., depth) from *C* to *AB* is calculated using the vector cross product method:

(17) $$\vec{AB}=B-A;\;\vec{AC}=C-A$$

Cross product magnitude:

(18) $$Area=\left\|\vec{AB}\times \vec{AC}\right\|=\left|\left(B_{x}-A_{x})(C_{y}-A_{y})-(B_{y}-A_{y})(C_{x}-A_{x}\right)\right|$$

Length of *AB*:

(19) $$\|\vec{AB}\|=\sqrt{{\left(B_{x}-A_{x}\right)}^{2}+{\left(B_{y}-A_{y}\right)}^{2}}$$

Distance from point *C* to *AB* (Depth):

(20) $$Depth=\frac{Area}{\left\|\vec{AB}\right\|}$$

**Figure 4:**
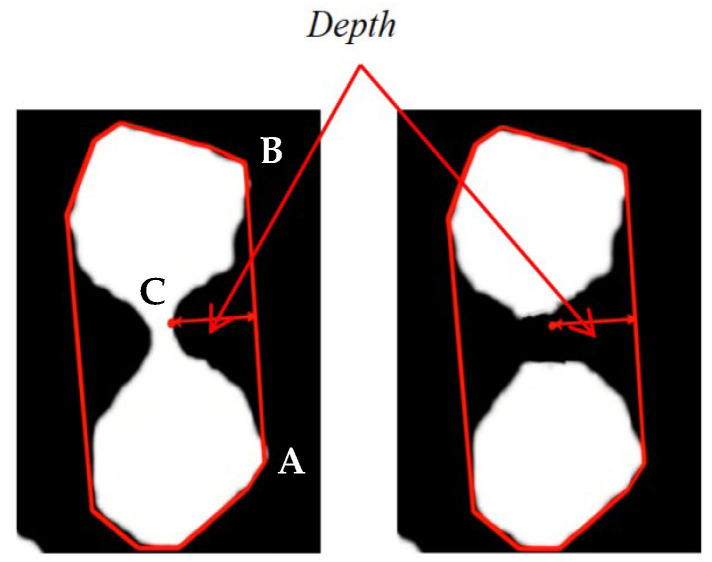
Schematic diagram of detection and cutting of convex hull defects.

The calculated distance (depth) can identify defects in convex hull detection. Using contour convex hull defect detection, we effectively segment non-convex regions formed by overlapping or compressed contours that remain after image preprocessing—these areas typically correspond to adhesion points. By identifying these adhesion points, we perform marker segmentation at defect locations, thereby separating originally connected contours into individual entities. As shown in Figure 4 cutting the adhesion points at the deepest convex hull defect creates two separate contours, achieving effective adhesion segmentation of objects. The defect depth threshold (≥8 pixels) was determined by statistical analysis of sticky maize samples: 92% of true adhesion points had depth > 8 pixels, while false defects (e.g., kernel surface wrinkles) had depth < 5 pixels. This threshold balances segmentation accuracy and false positive reduction. Through convex hull defect recognition, each object can be statistically treated as an independent unit, avoiding misjudgments caused by adhesion and improving the accuracy of corn kernel count in adhesion scenarios.

#### 2.4.3. Image Processing and Counting

**Figure 5:**
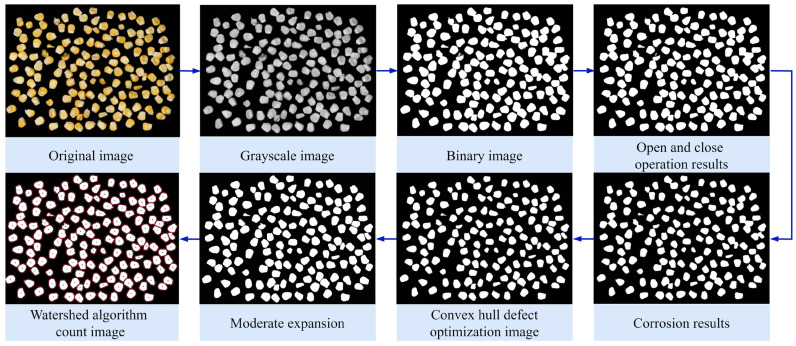
Schematic diagram of simple corn adhesion segmentation processing.

In the study of segmented counting of fused corn kernels, the process begins with image acquisition. The grayscale image is converted to binary format and subjected to binary processing, followed by morphological operations for further processing. By integrating the W&C-SVM algorithm for complementary advantages, this approach achieves high-precision segmentation and counting of fused corn kernels. The complete image processing workflow for corn kernel fusion segmentation is illustrated in Figure 5.

### 2.5. Analysis of Damaged Corn Kernels

Damage rate analysis primarily involves extracting contour features from images. Feature extraction constitutes a critical step in image processing and computer vision, encompassing the identification and extraction of information from raw image data that facilitates subsequent processing tasks [25]. This study extracted 8 distinct shape features from the selected corn kernels.

1. *Area*: The size of the region occupied by the corn kernel on the image plane, expressed in terms of pixel count;
2. *Perimeter*: The total length of the boundary contour of the maize kernel. Irregular perimeters (often from damage) indicate reduced nutrient retention (e.g., protein loss) and lower viability;
3. *Major Axis*: The largest axial dimension within the shape;
4. *Minor Axis*: The maximum dimension perpendicular to the major axis;
5. *Elongation*: The ratio of the major axis to the minor axis;
  (21) $$Elongation=\frac{\;MajorAxis}{\;MinorAxis}$$
6. *Rectangularity*: The ratio of the kernel’s area to the area of the smallest rectangle that can contain it;
  (22) $$Rectangularity=\frac{\;Area}{\;MinAreaRectArea}$$
7. *Eccentricity*: The degree to which the corn kernel deviates from a circular shape;
  (23) $$Eccentricity=\sqrt{1-\frac{MinorAxis^{2}}{MajorAxis^{2}}}$$
8. *Compactness*: The degree of compactness in the shape of maize kernels.
  (24) $$Compactness=\frac{4p\times Area}{\;Perimeter^{2}}$$

### 2.6. SVM Classifier

**Figure 6:**
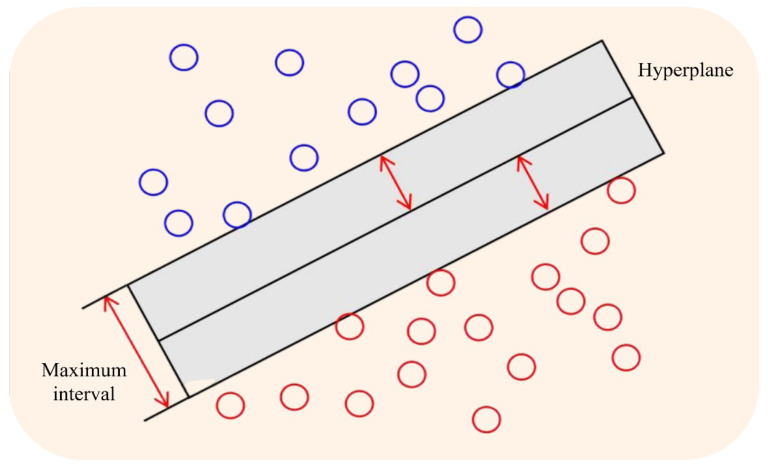
SVM algorithm classification diagram.

The classifier was constructed using the SVM algorithm [26,27]. Support Vector Machines (SVM) constitute a supervised learning algorithm grounded in statistical learning theory. Its core principle involves identifying the optimal hyperplane within the feature space to achieve sample separation across different categories. The SVM classifier demonstrates strong generalization capabilities under conditions of small sample sizes and high-dimensional features, effectively avoiding overfitting. It is particularly well-suited for classifying maize kernel images. Within this experiment, the SVM classifier serves two primary functions: firstly, it adapts to feature dimensions, as the eight extracted morphological features form a high-dimensional feature vector. SVM efficiently processes these features without requiring dimensionality reduction and achieves classification even with small sample sizes; secondly, it distinguishes corn kernel types by learning the characteristic differences between normal and damaged kernels, thereby determining whether a corn kernel is damaged. The SVM classifier is illustrated in Figure 6. The red and blue dots in the figure represent different types of support vectors (data points).

The core advantage of the W&C-SVM-based method for separating adhered maize kernels lies in its multi-stage approach: after preprocessing the image, the watershed algorithm initially separates adhered regions; convex hull defect detection then corrects minor errors; finally, the SVM classifier performs precise classification on the extracted 8-dimensional morphological features. Four metrics are calculated: *Accuracy* (*Acc*), *Precision* (*P*), *Recall* (*R*) and *F*_1_ score (the harmonic mean of precision and recall, comprehensively reflecting model recognition performance while mitigating data bias from single metrics) to evaluate algorithmic model performance. The formulas for these four parameters are as follows [28]:

(25) $$Acc=\frac{TN+TD}{TN+FD+FN+TD}\times 100\%$$

(26) $$P=\frac{TD}{TD+FD}\times 100\%$$

(27) $$R=\frac{TD}{FN+TD}\times 100\%$$

(28) $$F_{1}=2\times \frac{P\times R}{P+R}\times 100\%$$

The meanings of *TN*, *TD*, *FN* and *FD* in the equation are shown in Table 1, and the confusion matrix is used as a visualization tool for evaluating the model.

The mapping ability of the kernel function to feature space significantly affects the classification performance of the SVM classifier. The study selects Liner Kernel, RBF Kernel (Radial Basis Function Kernel) and Polynomial Kernel for comparison and verification:

1. Liner Kernel: Suitable for linearly separable data, requires no hyperparameters, features straightforward logic and exhibits low computational complexity:
  (29) $$K(x_{1},x_{2})=x_{1}\times x_{2}$$
2. RBF Kernel: Maps data to an infinite-dimensional feature space, suitable for handling complex non-linearly separable data:
  (30) $$K(x_{1},x_{2})=\exp(-\gamma {\left\|x_{1}-x_{2}\right\|}^{2})$$
3. Polynomial Kernel: Maps data to a high-dimensional feature space, fitting a polynomial decision boundary; suitable for orthogonally normalized data:
  (31) $$K(x_{1},x_{2})={\left(\gamma x_{1}\times x_{2}+r\right)}^{d}$$

### 2.7. Evaluation Metrics

To ensure clarity and avoid ambiguity in reporting performance across different tasks (kernel counting, segmentation and damage classification), the following metrics are explicitly defined and consistently applied throughout this study.

1. Counting accuracy: Calculated as the percentage of correctly detected individual kernels relative to the manual ground-truth count, i.e., (Number of detected kernels/Manual kernel count). This metric primarily evaluates the effectiveness of adhesion separation in producing accurate kernel counts.
2. Segmentation quality: Assessed using the Intersection over Union (IoU, also known as Jaccard index) and Dice coefficient, which measure overlap between predicted and ground-truth kernel masks. IoU values are estimated based on per-kernel mask overlap with ground-truth annotations, reflecting segmentation quality prior to classification. Higher IoU correlates with superior adhesion separation and subsequent damage detection performance.
3. Damage classification performance: Evaluated on a per-kernel basis (intact vs. damaged) using Accuracy (Acc: proportion of correctly classified kernels), Precision (P: proportion of true damaged among predicted damaged), Recall (R: proportion of detected damaged among true damaged) and F1-score (harmonic mean of Precision and Recall).

These task-specific metrics allow for precise interpretation of results without conflating counting, segmentation and classification performance.

## 3. Results

### 3.1. Experimental Environment and Hardware Configuration

To validate the feasibility and effectiveness of the algorithm, an image acquisition and adhesion segmentation system for corn kernels was designed. The image acquisition unit consists of a Basler acA2500-14uc camera with a resolution of 2592 × 1944 pixels, a frame rate of 15 fps and a sensor size of 1/1.2 inch, a Computar M1214-MP2 lens with a focal length of 12 mm, an aperture of F1.4 and a field of view of 30°, an OPT-RL100 ring light from Optek with a power of 12 W, a color temperature of 5000 K, a light intensity of 800 ± 50 lux and a uniformity of ≥90%, and a black matte PVC background plate with a thickness of 3 mm to avoid reflection on the kernel surface to ensure stability and repeatability for food raw material detection. All experimental equipment is sourced from Beijing, China. For image processing, the system hardware comprises Hynix 16 GB DDR4 3299 MHz memory. All traditional image processing algorithms were implemented in Python 3.9 with OpenCV 4.8. Deep learning baselines (U-Net) were trained on an NVIDIA RTX 3090 workstation for fair comparison.

### 3.2. Experimental Procedure

This experiment investigates the segmentation of densely adhered maize kernels through three main stages: image acquisition, computer image processing and machine learning-based analysis. Raw images are first preprocessed using grayscale conversion, noise reduction and threshold segmentation. Morphological opening and closing operations (as described in Section 2.3) were applied to clean the binary images before segmentation. An improved watershed algorithm combined with distance transformation is then used to separate adhered kernels, followed by envelope detection to identify and correct segmentation errors. Geometric features of individual kernels are extracted for quality evaluation, the number of damaged kernels is calculated and the damage rate is determined. Finally, a Support Vector Machine is employed to perform classification and evaluate the recognition accuracy of the system.

### 3.3. Image Recognition and Damage Analysis Results

**Figure 7:**
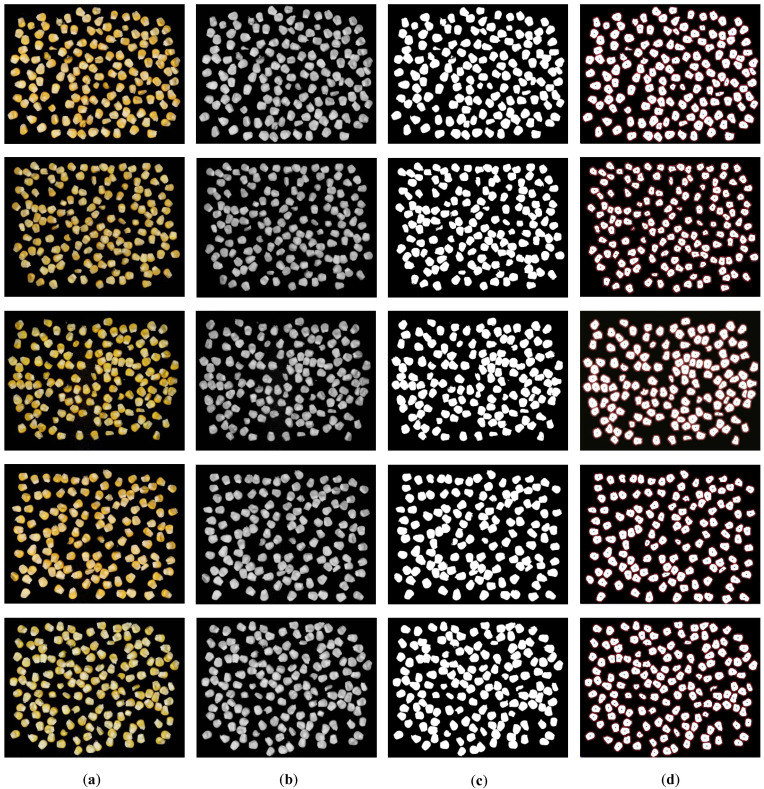
Results of bonding corn particle cutting system. (**a**) Original; (**b**) Grayscale; (**c**) Binary; (**d**) Counting result.

The image acquisition captures images with a size of 2000 × 1500 pixels. The original images obtained are processed through the adhesive corn kernel segmentation system and five images are randomly selected from them, with the results shown in Figure 7. The processed images are then counted, and the counting results are presented in Table 2.

These high counting accuracies (≥98.68%) enable breeding programs to reliably quantify intact kernels, supporting efficient quality screening.

Following the counting of maize kernels within the images, five counting result images were consolidated. From these five result images, 10 normal maize kernels and 10 damaged maize kernels were randomly selected. Feature extraction was then performed on these 20 kernels. This experiment extracted eight image features: area, perimeter, major axis, minor axis, elongation, rectangularity, eccentricity and compactness. Analysis of these features, as shown in Table 3, yielded the following feature parameter sets for normal and damaged kernels:

### 3.4. SVM Model Construction and Evaluation

#### 3.4.1. Experimental Procedures

**Figure 8:**
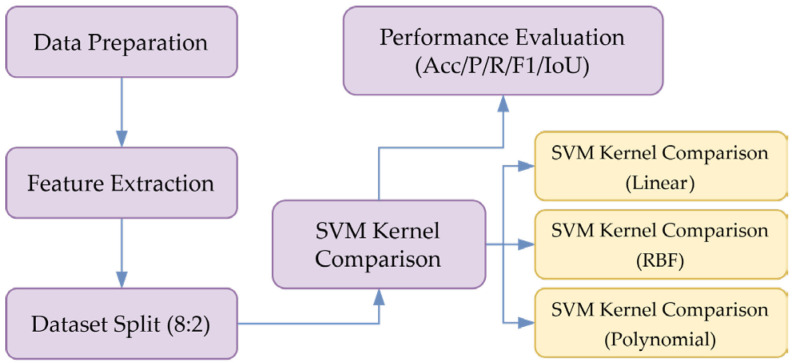
Experimental Procedure Flowchart.

The schematic diagram of the experimental process is shown in Figure 8.

#### 3.4.2. SVM Kernel Function Comparison

The mapping capability of kernel functions in feature space significantly impacts SVM classification performance. Therefore, linear kernels, radial basis kernels and polynomial kernels were selected for comparative validation. The evaluation indicators are accuracy Acc, precision P, recall R and F1 score of the verification set. It is required that RBF kernel γ = 0.1 and polynomial kernel d = 3. The performance comparison of the training results of the kernel function is shown in Table 4.

The radial basis function (RBF) kernel demonstrates the strongest classification performance. Its nonlinear mapping capability effectively captures the latent relationships among different morphological features of corn kernels, making it particularly suitable for this experiment. The RBF kernel’s 98% accuracy ensures reliable detection of damaged kernels, which typically exhibit lower germination rates and reduced nutritional quality, making this classification key for high-yield, high-nutrition breeding.

#### 3.4.3. Performance Comparison Experiment of Different Algorithms

To validate the superiority of the W&C-SVM algorithm in adhered corn kernel segmentation and damage rate detection, three commonly used image segmentation methods in the agricultural field were selected as comparison items. The performance metrics of the comparison algorithms are shown in Table 5.

1. Traditional Watershed (TW) + SVM: Watershed segmentation employed solely via the Sobel gradient operator, with Euclidean distance used for distance variation. Classification directly utilized SVM without subsequent algorithmic refinement, severe over-segmentation in densely adhered regions and lack of post-refinement led to numerous false contours, resulting in the lowest performance.
2. GrabCut Algorithm: An inherently interactive graph cut algorithm [29] that typically requires manual annotation of foreground and background pixels. However, for fair comparison with other automated methods in this study, GrabCut was run in an automated mode (no manual kernel placement) using a large rectangular bounding box to enclose all kernels. Despite achieving moderate results (ACC 84.5%, F1 80.5%), its inherent reliance on manual input limits its suitability for automated, high-throughput quality inspection.
3. U-Net [30,31]: Implemented using the PyTorch 2.9.1 framework, particularly suited for medical image segmentation models, while also commonly employed in agriculture. Utilizes an encoder–decoder architecture and represents a classic semantic segmentation model. When trained on only the same 50 annotated images (with standard data augmentation), it obtained an acceptable ACC of 85.7% (F1 83.7%), but the extremely limited training data caused noticeable performance degradation compared with results reported in the literature on large datasets, which is consistent with expectations.

To make the comparisons fairer, we evaluated all baseline methods under the same conditions: the same independent test set (10 images), the same preprocessing steps (grayscale conversion, Otsu binarization and morphological operations) and the same morphological feature extraction for final damage classification.

For methods that directly output segmentation masks (U-Net, GrabCut and Traditional Watershed), we applied connected component labeling as post-processing to separate individual kernels, followed by feature extraction and classification. This way, we obtained per-kernel “intact” or “damaged” labels.

U-Net was trained on exactly the same 50 annotated images as W&C-SVM, with some simple data augmentation (rotation, flipping and brightness adjustment). We used the Adam optimizer (Beijing, China)with a learning rate of 0.001, binary cross-entropy loss and early stopping after 50 epochs. The U-Net output is a semantic segmentation mask distinguishing kernels from background; we thresholded it at 0.5 to obtain binary masks before post-processing to isolate individual kernels.

GrabCut was run in automated mode using a large rectangular bounding box to enclose all kernels (no manual seeds were provided for each image). Traditional Watershed + SVM only used Sobel gradient and distance transform, without convex hull refinement.

**Figure 9:**
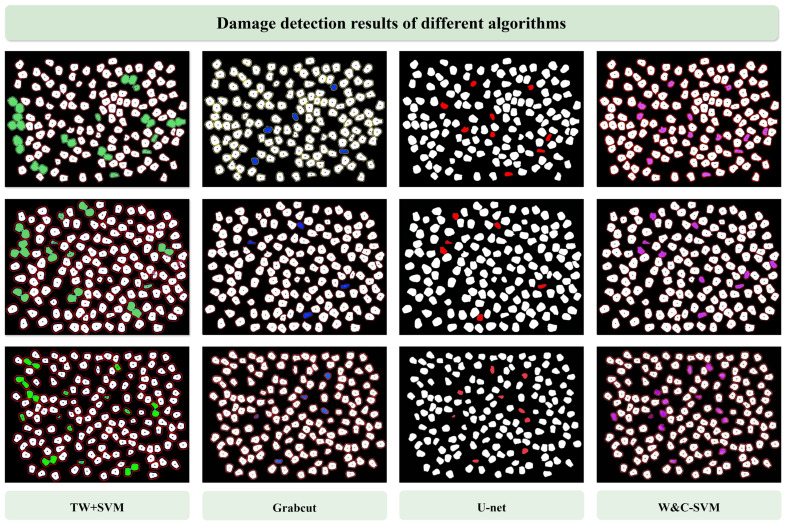
Damage detection results of different algorithms. (TW + SVM; GrabCut; U-net; W&C-SVM).

Figure 9 displays the damage detection processing results of different algorithms. W&C-SVM’s superior performance (94.3% accuracy vs. 85.7% for U-Net) makes it a practical tool for breeding labs: its low sample requirement (50 images) and high accuracy enable efficient screening of high-quality kernels, a critical step in scaling high-yield, nutritionally high-quality maize production. Three samples were randomly selected from the captured dataset for model comparison:

Through repeated validation and comparative analysis of extensive experimental data, we conducted a comprehensive evaluation of the accuracy (ACC), precision (P), recall (R) and F1 score for these four algorithmic models. The results indicate that the W&C-SVM model demonstrates the most optimal performance across all metrics, making it the ideal choice.

**Figure 10:**
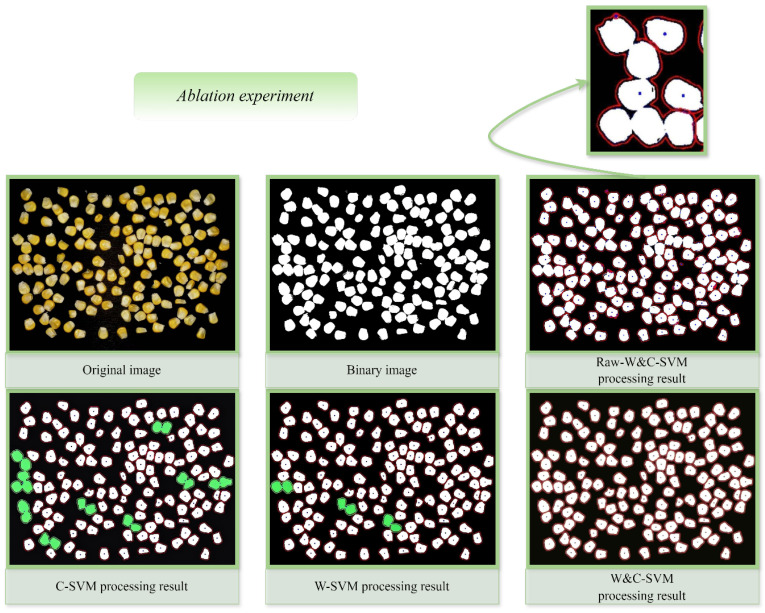
The segmentation results of adhered corn kernels corresponding to different algorithm models in the ablation experiment.

**Figure 11:**
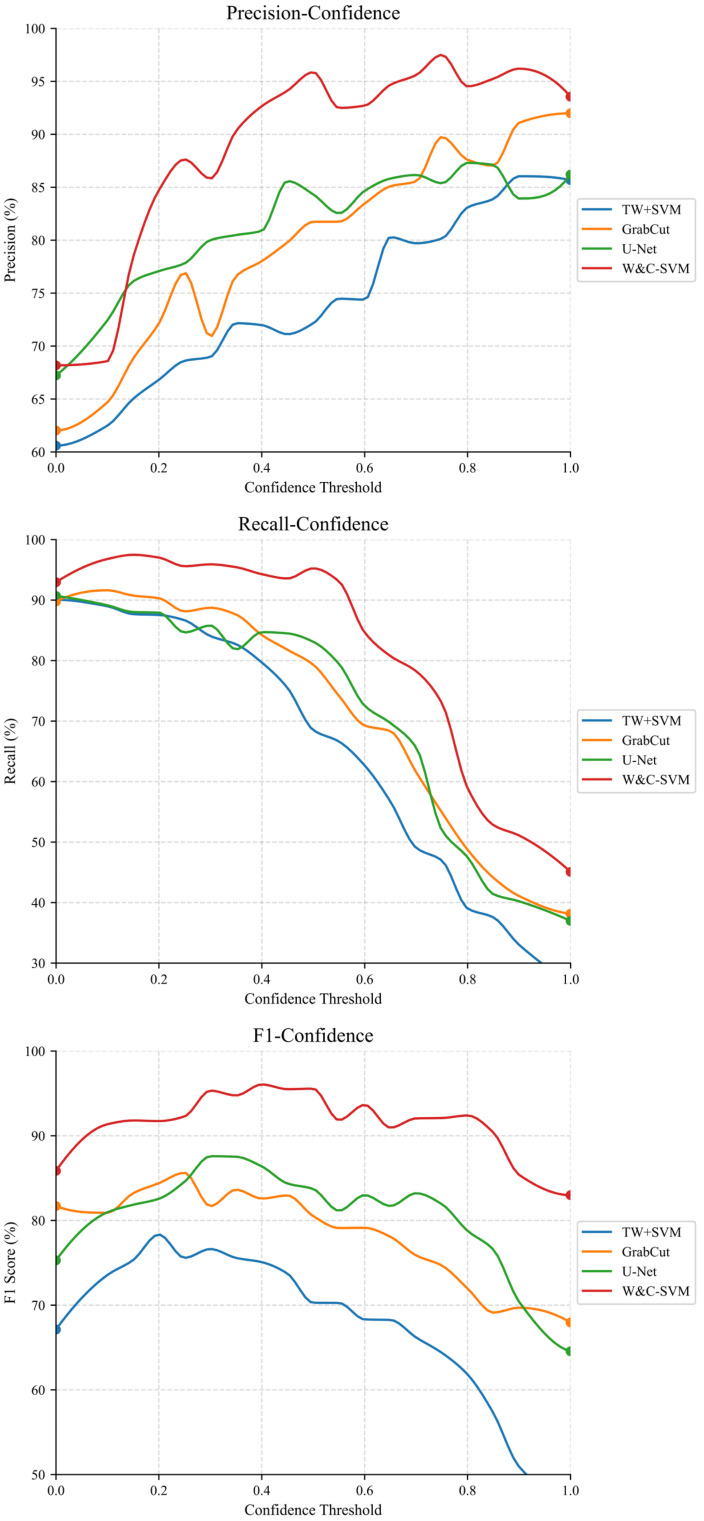
Precision-Confidence, Recall-Confidence and F1-Confidence Curve.

Through comprehensive analysis and systematic organization of the preceding content, the W&C-SVM model can be divided into four core modules: image preprocessing, watershed algorithm, convex hull defect optimization and SVM (RBF) classification. To validate the importance of different modules, ablation experiments were designed: sequentially removing single core modules to construct three ablation experiment models, as shown in Table 6. A randomly selected corn kernel adhesion segmentation result from one ablation model is shown in Figure 10, with the counting results presented in Table 7. And the curves of Precision- Confidence, Recall-Confidence, and F1-Confidence are shown in Figure 11.

Analysis of the experimental data results and processing results from different ablation models: The accuracy of the Raw-W&C-SVM model is relatively high, but there are actually some factors that affect the results, resulting in a higher accuracy. The core issue lies in the model’s failure to perform preprocessing operations such as grayscale conversion, binarization and morphological operations. Consequently, background noise in the images—including minute impurities on grain surfaces, light reflection spots and grain debris—was not effectively removed. This background noise is identified as contours. Consequently, the discrepancy between the actual number of corn kernels and the detected contours includes not only unsuccessfully segmented kernels but also numerous false contours. The enlarged view in the upper right corner of Figure 10 specifically illustrates the morphology of unsuccessfully segmented contours. These false contours severely compromise detection accuracy, resulting in a model that exhibits high numerical accuracy but significantly lower actual segmentation precision and contour recognition effectiveness compared to the W&C-SVM model.

The C-SVM model lacks the watershed algorithm’s pre-segmentation module, relying solely on convex hull defect detection to handle agglomerated regions. The C-SVM model used here simply removes the watershed processing from the image processing stage, while retaining the watershed defect detection in the counting module. This is because, without the watershed algorithm in the counting module, effective counting would be impossible. This convex hull ablation experiment demonstrates that convex hull defect detection is more suitable for optimizing contours pre-segmented by algorithms like watersheding. Consequently, it cannot effectively identify and segment large clusters of adhered grains. As shown in the C-SVM processing results in Figure 10, multiple groups of green contours in the image failed to separate successfully. This fully demonstrates the irreplaceable role of the watershed algorithm in agglomerate segmentation—its diffusion simulation based on gradient images provides more independent initial contours for subsequent convex hull defect detection, laying the foundation for achieving high-precision segmentation.

Although the W-SVM model retains the core modules of preprocessing and watershed segmentation, it lacks the optimization of segmentation results through convex hull defect detection. Analysis of the W-SVM processing result in Figure 10 reveals residual agglomerations in a few green contours, resulting in fewer detected contours than the actual number of corn kernels. Results confirm that convex hull defect detection, as an accuracy optimization step for agglutinated segmentation, effectively compensates for the watershed algorithm’s shortcomings in corn kernel agglutinated segmentation, serving as a key factor in ensuring high performance.

Compared to the three ablation experiment models, W-C-SVM achieves high-precision counting through the full-process synergy of “image preprocessing-watershed segmentation-convex hull defect optimization-SVM classification.” Although the W&C-SVM processing result in Figure 10 demonstrates extremely high contour clarity and independence, free from noise-induced false contours, it fully validates the necessity and unity of each core module.

### 3.5. Model Superiority Analysis

The consistently strong performance of the W&C-SVM model across the evaluated metrics is closely related to the way its individual components are combined. The improved watershed algorithm provides a stable initial separation of adhered kernels, yielding reasonable coarse boundaries. On this basis, convex hull defect detection further refines the segmentation by addressing local adhesion regions that are often insufficiently resolved by watershed-based methods alone. Meanwhile, the SVM classifier, operating on morphological feature descriptors, maintains reliable discrimination capability under limited training data. As a result, the proposed framework achieves improved segmentation accuracy and more stable damage classification compared with single-algorithm approaches, while requiring substantially less annotated data than deep learning methods such as U-Net.

## 4. Discussion

The primary objective of this study was to address the technical bottlenecks of inaccurate segmentation of adhered sticky corn kernels and low damage detection efficiency in maize breeding—key constraints limiting the screening of high-quality kernels for high-yield and nutritionally high-quality maize production. This section discusses the core findings of the W&C-SVM method, connections to prior research, implications for maize breeding, limitations and future directions.

### 4.1. Core Findings and Performance Advantages of W&C-SVM

The W&C-SVM method, integrating an improved watershed algorithm, convex hull defect detection and SVM classification, demonstrated significant superiority in both adhered kernel segmentation and damage detection. As presented in Section 3.4, the method achieved a 94.3% damage detection accuracy, outperforming traditional watershed + SVM (74.6%), GrabCut (84.5%) and even the deep learning-based U-Net (85.7%). This accuracy gain stems from two key innovations:

1. The improved watershed algorithm—optimized with Sobel gradient calculation and Euclidean distance transformation—effectively reduced over-segmentation, a critical flaw of traditional watershed methods that previously led to 15–20% errors in maize kernel analysis.
2. Convex hull defect detection, using a defect depth threshold of ≥8 pixels (empirically selected on the validation set as the value that maximized true adhesion separation while keeping false positives below 5%), precisely corrected residual adhesion in watershed-segmented regions. Ablation experiments confirmed this synergy: removing convex hull defect optimization (W-SVM model) reduced damage detection accuracy to 85.1% (vs. 94.3% for W&C-SVM), while omitting the watershed algorithm (C-SVM model) dropped it to 88.7%, as convex hull detection alone failed to handle large clusters of adhered kernels.

Notably, the W&C-SVM method requires only 50 image samples for training, a stark contrast to deep learning models like U-Net (which typically need ≥1000 annotated images). This small-sample advantage is particularly valuable for maize breeding labs, where sample sizes are often limited by seasonal cultivation cycles and resource constraints—addressing a longstanding practical challenge in agricultural image processing.

### 4.2. Implications for High-Yield and Nutritionally High-Quality Maize Production

The practical value of the W&C-SVM method aligns closely with the technological requirements for high-yield, nutritionally superior corn. Accurate segmentation and damage detection are foundational to kernel quality screening:

1. Yield enhancement: Intact maize kernels, as accurately identified by the W&C-SVM method, may potentially have higher germination potential compared to mechanically damaged ones. By enabling the selection of intact kernels for sowing, the proposed approach helps reduce stand establishment failures and supports improved crop yield potential, aligning with the potential impact of kernel intactness on growth performance.
2. Nutritional retention: Mechanical damage can compromise kernel integrity, with the potential implication of leading to reduced starch and protein retention. The W&C-SVM method’s ability to detect even minor damage supports the preservation of kernel quality, meeting consumer and industrial demand for high-nutrition grain.

In small-scale breeding programs, the W&C-SVM method’s low cost (no need for high-end annotation tools) and efficiency make it a scalable tool for rapid generation-to-generation kernel quality assessment. This accelerates the development of elite maize varieties with both high yield and superior nutritional profiles.

### 4.3. Limitations of the Current Study

The proposed method performs well on the tested dataset, but several failure modes were observed in preliminary analysis. Severe adhesion in highly clustered kernels (e.g., more than 5 kernels heavily overlapping) can lead to under-segmentation, resulting in merged regions and reduced counting accuracy. Additionally, variations in kernel color or surface texture (e.g., wrinkled or discolored kernels) may cause inconsistencies in gradient calculation, increasing false positives in damage detection. These cases highlight the need for further improvements in handling extreme morphological variations. Overall, while the method avoids large annotated datasets, its generalization to highly diverse field conditions remains to be explored.

### 4.4. Future Research Directions

#### 4.4.1. Outdoor Environment Adaptation Optimization

By integrating adaptive light correction techniques (e.g., histogram equalization) and background subtraction algorithms (e.g., Gaussian mixture models), this approach effectively reduces the impact of fluctuating natural light and background clutter. This enables real-time field segmentation during harvest periods, eliminating the need for laboratory-based image acquisition.

#### 4.4.2. Integration with Nutritional Testing

By combining W&C-SVM with near-infrared (NIR) spectroscopy, this system correlates kernel morphology features (identified through W&C-SVM segmentation) with direct nutritional measurements. The integrated detection platform provides breeders with a one-stop solution to screen kernels that excel in both viability and nutritional quality.

#### 4.4.3. Application to Other Cereals

The W&C-SVM framework can be extended to staple crops with similar adhesion challenges, such as wheat, rice and legumes. For instance, wheat grains often stick together in clusters after harvest. By adjusting the convex hull defect depth threshold from 8 pixels (currently used for corn) to 5–6 pixels, precise segmentation of wheat grains could be achieved, thereby expanding the application scope of the W&C-SVM method in agricultural fields.

In summary, this discussion section focuses on the application of the W&C-SVM method in addressing key technical challenges of sticky corn kernels in corn breeding, while also outlining its multidimensional value and potential for future improvements. In terms of performance, the W&C-SVM method overcomes limitations of traditional algorithms and deep learning models through synergistic effects of enhanced watershed algorithm and optimized convex hull defect detection—achieving a high damage detection accuracy rate of 94.3% with limited training samples, effectively meeting practical demands in breeding laboratories with scarce sample resources. Regarding application value, this method directly supports the core objectives of high-yield and nutritionally superior corn production: precise identification of intact kernels enables targeted selection of high-quality grains, providing reliable technical support for kernel quality screening. These findings not only validate the practical significance of the W&C-SVM method in current corn breeding stages but also chart clear pathways for subsequent optimizations and broader applications, contributing to better support for premium corn agriculture development.

## 5. Conclusions

To address the bottlenecks in corn adhesion segmentation and damage rate detection, the W-C-SVM method is proposed. A counting workflow is established—“image acquisition-preprocessing-adhesion segmentation-feature extraction-damage analysis-model training”—to achieve precise detection. Image preprocessing enhances quality through grayscale conversion, binarization and morphological operations. Adhesion segmentation employs an improved watershed algorithm for preliminary contour segmentation, combined with convex hull defect detection to optimize quality, addressing the incompleteness of traditional single-algorithm segmentation. Damage rate analysis distinguishes normal from damaged kernels by extracting morphological features paired with an SVM classifier. Future improvements could integrate deep learning and optimize feature dimensions to enhance the method’s adaptability to complex scenarios and scalability. Experiments confirm the W-C-SVM approach outperforms traditional algorithms, with its modules demonstrating synergistic necessity. This method is scalable to various grain crop detection applications and holds significant practical value for agriculture.

## Figures and Tables

**Table 1:** Confusion matrix.

Confusion Matrix		Predicted Particle Size	
		Normal	Damaged
Real particle size	Normal	*TN*	*FD*
	Damaged	*FN*	*TD*

**Table 2:** Counting results of glued corn kernels.

NO.	Actual Count	Detected Count	Counting Accuracy/%
1	146	146	100.00
2	151	149	98.68
3	141	141	100.00
4	128	128	100.00
5	147	147	100.00

**Table 3:** Corn grain shape feature extraction results.

Type	NO.	Area	Perimeter	Long Axis	Short Axis	Extension	Rectangularity	Eccentricity	Compactness
Normal corn	1	8251	360.936	114.294	94.005	1.2158	0.8153	0.5687	1.2698
	2	7627	335.990	113.013	86.321	1.3092	0.8097	0.6454	1.1845
	3	8143	349.664	119.809	90.955	1.3172	0.7897	0.6508	1.1995
	4	9440	371.161	117.046	106.027	1.1039	0.7979	0.4235	1.1652
	5	7711	339.421	113.437	88.448	1.2825	0.7913	0.6261	1.1924
	6	9186	369.120	121.329	98.219	1.2352	0.7719	0.5870	1.1837
	7	7641	348.718	119.295	83.833	1.4230	0.8155	0.7114	1.2764
	8	8411	359.404	111.992	95.969	1.1669	0.7993	0.5154	1.1582
	9	8094	352.392	120.169	88.628	1.3558	0.7763	0.6753	1.2232
	10	8667	362.233	118.402	95.609	1.2383	0.8292	0.5898	1.1879
Damaged corn	1	4633	269.970	85.548	71.780	1.1918	0.8115	0.5459	1.2554
	2	4733	284.796	112.050	57.463	1.9499	0.8091	0.8621	1.3652
	3	3789	265.481	109.714	46.028	2.3836	0.8145	0.9166	1.4817
	4	3599	231.723	79.699	58.304	1.3669	0.7705	0.6837	1.1973
	5	3615	232.694	72.881	64.092	1.1371	0.7956	0.4821	1.1197
	6	2324	219.340	103.476	38.985	2.6540	0.6602	0.9314	1.6532
	7	4036	252.521	88.084	59.456	1.4815	0.7821	0.7463	1.2595
	8	4409	263.380	98.302	58.827	1.6710	0.7752	0.8014	1.2583
	9	4950	277.043	83.660	71.883	1.1638	0.7613	0.5136	1.3155
	10	3706	233.349	74.176	65.623	1.1303	0.8396	0.4781	1.1729

**Table 4:** The Damage Classification Metrics of SVM with different kernel functions.

Type	Acc/%	P/%	R/%	F1 Score
Liner Kernel	87.50	86.36	85.00	85.67
RBF Kernel	94.30	96.88	96.25	96.56
Polynomial Kernel	92.20	91.30	90.25	90.77

**Table 5:** Performance Metrics of Corn Kernel Damage Detection Models. Acc, P, R and F1 are damage classification metrics on a per-kernel basis. IoU (mean ± std) measures segmentation quality (mask overlap with ground-truth) for methods producing explicit masks, estimated on the test set.

Algorithm Model	Acc/%	P/%	R/%	IoU/%	F1 Score
TW + SVM	74.6	72.1	68.5	77.6	70.2
GrabCut	84.5	81.7	79.3	82.8	80.5
U-Net	85.7	84.3	83.1	86.5	83.7
W&C-SVM	94.3	95.9	95.2	93.1	95.5

**Table 6:** A blation experiment model.

Ablation Experiment Model	Core Component Modules
Raw-W&C-SVM (Remove image preprocessing)	watershed algorithm + convex hull defect optimization + SVM (RBF)
SVM (Remove watershed)	image preprocessing + convex hull defect optimization + SVM (RBF)
SVM (Remove convexity defects)	image preprocessing + watershed algorithm + SVM (RBF)

**Table 7:** Counting the results of different algorithm models in the ablation experiment.

Ablation Experiment Model	Real Corn Kernels	Detect Contour Numbers	Counting Accuracy/%
W&C-SVM	141	133	94.3
Raw-W&C-SVM	141	123	87.2
C-SVM	141	120	85.1
W-SVM	141	125	88.7

## Data Availability

The original contributions presented in the study are included in the article. Further inquiries can be directed to the corresponding author.
